# Optimizing antibody expression by using the naturally occurring framework diversity in a live bacterial antibody display system

**DOI:** 10.1038/srep17488

**Published:** 2015-12-03

**Authors:** T. Noelle Lombana, Michael Dillon, Jack Bevers III, Christoph Spiess

**Affiliations:** 1 Department of Antibody Engineering, Genentech Research and Early Development, 1 DNA Way, South San Francisco, CA, 94080, USA

## Abstract

Rapid identification of residues that influence antibody expression and thermostability is often needed to move promising therapeutics into the clinic. To establish a method that can assess small expression differences, we developed a Bacterial Antibody Display (BAD) system that overcomes previous limitations, enabling the use of full-length formats for antibody and antigen in a live cell setting. We designed a unique library of individual framework variants using natural diversity introduced by somatic hypermutation, and screened half-antibodies for increased expression using BAD. We successfully identify variants that dramatically improve expression yields and *in vitro* thermostability of two therapeutically relevant antibodies in *E. coli* and mammalian cells. While we study antibody expression, bacterial display can now be expanded to examine the processes of protein folding and translocation. Additionally, our natural library design strategy could be applied during antibody humanization and library design for *in vitro* display methods to maintain expression and formulation stability.

Good functional expression is a hallmark in the successful path of a protein therapeutic. While antibodies can be produced in gram/liter quantities in mammalian or *Escherichia coli* (*E. coli*) cells, efforts to improve yields are typically required to reach manufacturing levels. Screening methods to improve antibody expression may be applied at multiple stages in therapeutic development, and therefore, use of an *E. coli* expression host for this optimization is attractive as growth and expression is faster than in a mammalian host.

Several approaches have been taken to increase antibody yields in *E. coli*, including changes of the signal sequence, translation initiation region, and cell strain, as well as co-expression of chaperones^1^,^2^,^3^,^4^. Additionally, saturation mutagenesis of select positions in the antibody sequence has been shown to identify mutants conferring superior functional expression and stability^5^. However, as this approach predominantly leads to amino acid changes that are not found in the natural repertoire of antibodies, a risk of introducing immunogenic sequences into antibodies of therapeutic interest remains.

To reduce this risk we investigated the natural antibody diversity within a subgroup that occurs during somatic hypermutation. We reasoned that these changes are structurally desired and tolerated by the immune system, since the resulting antibody is productively secreted by B-cells. Moreover, we focused our studies on the framework regions, and not the complementarity determining regions, to maintain the antibody affinity. While this concept to create natural diversity has been used by others to generate libraries for antibody discovery or affinity maturation^6^, its contribution to antibody folding and conformational stability is poorly understood.

Screening and detection of small changes in antibody expression is a challenging problem. Western blot, ELISA, or small scale purification have been used, but these techniques are limited by their inherent variability and low throughput. These problems could be overcome using an antibody display system, as it enables high throughput screening of large libraries. Several cell display technologies have been developed over the past 20 years. While the fast replication times of bacteria should offer bacterial display accelerated round to round progression compared to other cell display technologies^7^,^8^,^9^, these advantages have been offset by significant compromises in order to retain antibody in the cell and keep it accessible to antigen^10^. Displaying large molecules as fusions to outer membrane proteins is difficult, limiting the applications of these systems to peptides or small proteins^11^,^12^,^13^. To allow display of larger proteins, systems were developed to bring ligands into the *E. coli* periplasm. While this was successful for small ligands^14^,^15^, large antigens required removal of the outer membrane^16^, which leads to cell death and requires additional time-consuming molecular manipulation between rounds. As a result, these systems have not been widely adopted. To overcome these limitations, we developed a live Bacterial Antibody Display (BAD) system in *E. coli*, and applied it to study antibody expression and folding, a novel application for cell display.

In our BAD system, the antibody is exported into the *E. coli* periplasm to facilitate correct folding. Using an engineered cell strain with an *lpp* gene deletion, and with the addition of EDTA, we enable fluorescently labeled antigen to enter these cells and bind expressed antibody. This is the first time a large protein antigen has been shown to enter and be retained in a live bacterial cell setting. These stained cells can be sorted by FACS and recovered for rapid round to round progression. Furthermore, with no tether needed, we can display a variety of antibody formats: full-length IgG, knobs-into-holes half-antibodies, or Fab.

In this work, we show that BAD has very high resolution and can resolve small differences in antibody functional expression. These properties allowed us to identify framework variants that substantially increase expression and *in vitro* thermostability of two therapeutically relevant antibodies, anti-IL-13 and anti-VEGF. Furthermore, the variants identified in *E. coli* translate to improved expression in a mammalian host system. In summary, we establish BAD as a valuable technology to study and improve *in vitro* properties of a protein.

## Results

**Figure 1:**
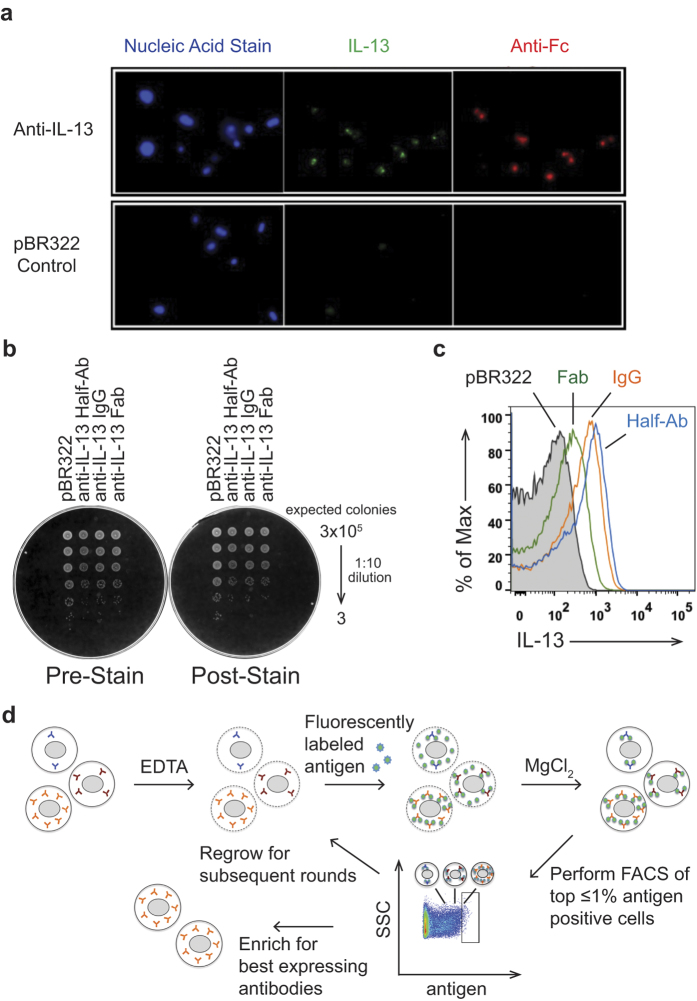
Antigen can be targeted to antibody expressing cells for Bacterial Antibody Display (BAD). (**A**) Fluorescence microscopy visualizes specific targeting of IL-13 antigen to antibody expressing cells. Cells expressing an anti-IL-13 antibody or the empty vector control (pBR322) were stained with Syto 41 (nucleic acid stain, blue), Alexa^488^ labeled IL-13 (green), or DyLight^649^ labeled anti-human Fc F(ab’)_2_ (red) antibodies. Only cells expressing the antibody stain positive with IL-13 antigen and anti-Fc. (**B**) Viability of cells is not impacted by the staining conditions. Serial dilutions of cells expressing different anti-IL-13 antibody formats or the empty control vector (pBR322) before (Pre-Stain) and after EDTA treatment and antigen staining, followed by MgCl_2_ addition (Post-Stain) were plated. No difference in viability is observed between the unstained and stained cells. (**C**) Flow cytometric analysis of cells expressing different formats of an anti-IL-13 antibody (IgG (orange), half-antibody (blue), and Fab (green)) or pBR322 control cells (black, shaded) after staining with fluorescently labeled IL-13. All formats are suitable for BAD with a shift above the pBR322 control. (**D**) Schematic diagram of BAD. Cells are permeabilized by treatment with EDTA, incubated with fluorescently labeled antigen, and the integrity of the outer membrane restored by addition of MgCl_2_. The top ≤ 1% antigen-positive cells are sorted by flow cytometry and either progress after regrowth into a subsequent round for further enrichment or are collected for analysis.

Existing bacterial display systems have technical limitations preventing the use of full-length antibody formats and large protein antigen together in a live cell setting^3^,^10^,^11^,^13^,^14^,^15^,^16^. To overcome these limitations, we explored novel ways to deliver the antigen past the bacterial outer membrane. We took advantage of the observation that deletion of Lpp, one of the major outer membrane proteins, renders the outer membrane semi-permeable, and that EDTA can further permeabilize the membrane^17^,^18^. While this genetic background is exploited to leak proteins from the periplasmic space into the media to facilitate purification^19^, we rationalized that the converse process could provide a mechanism to deliver proteins into the periplasm. To test this hypothesis, we expressed an anti-IL-13 IgG in a ∆*lpp* background and studied by fluorescent microscopy if cells treated with EDTA can bind exogenously added IL-13 cytokine that is fluorescently labeled. Only cells expressing antibody retain antigen (Fig. 1A), indicating that a membrane tether is not required to efficiently retain antibody in the cells. While IL-13 antigen has a molecular weight of ~15 kDa, staining with an anti-Fc F(ab’)_2_ suggests that antigens up to ~100 kDa can efficiently enter and be retained in the periplasm of permeabilized cells. However, we discovered that use of EDTA to permeabilize the cells resulted in decreased cell viability (data not shown). To overcome the viability decrease, we added MgCl_2_ after staining with antigen and EDTA, which restored the viability to levels seen before EDTA treatment (Fig. 1B).

Next, we explored if other antibody formats could be efficiently retained in the periplasm to bind antigen. Similar to the full-length antibody, both the Fab and half-antibody formats can be expressed and stained with antigen without impacting viability (Fig. 1B). While empty vector control (pBR322) cells show auto-fluorescence by FACS that contributes to a high background, all tested antibody formats give an antigen specific signal above this background that would enable efficient sorting (Fig. 1C). Half-antibodies with knobs-into-holes Fc technology are of interest as they can be used to generate full-length bispecific antibodies^20^,^21^,^22^. For all following display experiments, half-antibody constructs were utilized.

**Figure 2:**
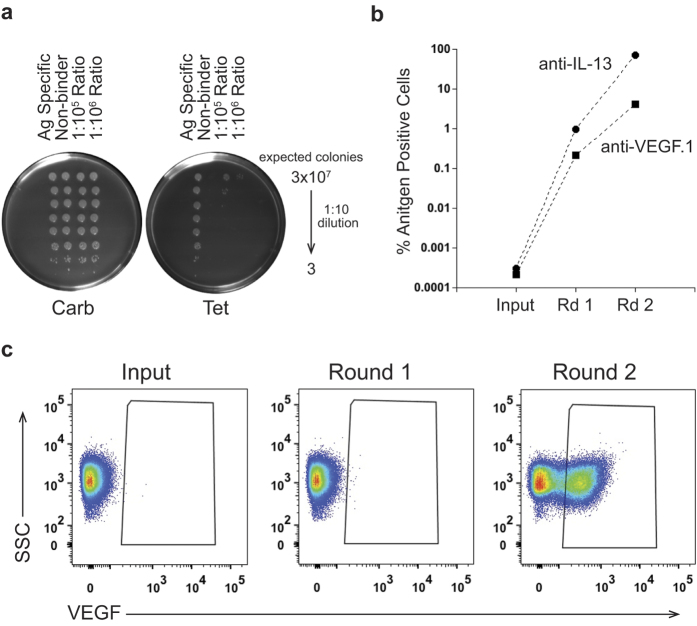
BAD can rapidly enrich rare binding antibodies from a pool of non-binders. (**A**) Bacteria carrying a plasmid encoding an anti-IL-13 antibody (carbenicillin (Carb) and tetracycline (Tet) resistant) were spiked 1:10^5^ or 1:10^6^ into bacteria transformed with a plasmid coding for an anti-VEGF.1 antibody (carbenicillin resistant). The existence of rare anti-IL-13 expressing bacteria was visualized by plating serial dilutions onto carbenicillin (total bacterial count) and tetracycline (bacteria carrying anti-IL-13 plasmid only) containing plates, confirming the expected ratios. (**B**) Spiked cultures were BAD sorted with Alexa^647^-labeled antigen. Sort input and outputs were plated on both a carbenicillin and a tetracycline plate, as in (**A**) to determine the ratio of antigen positive cells in the population. Rapid enrichment is seen of cells displaying an anti-IL-13 (circle) or anti-VEGF-antibody (square) spiked into a pool of cells displaying non-binding antibodies at a ratio of 1:10^6^ over two rounds (Rd) of BAD sorting. (**C**) Flow cytometric analysis of the anti-VEGF spiking experiment in Fig. 2B. A significant portion of the population binds to VEGF antigen after two rounds of FACS.

We have shown that our Bacterial Antibody Display (BAD) technology overcomes the current limits of bacterial display by delivering full-length antigen into live cells which should allow rapid round to round progression using FACS selection (Fig. 1D). In a first step we tested enrichment of a binding antibody from a pool of non-binders. We spiked cells expressing an anti-IL-13 antibody into a pool of cells expressing anti-VEGF.1 antibodies. To provide a means of tracking ratios of each antibody in the mixture, we used an anti-VEGF plasmid conferring only carbenicillin resistance, while the anti-IL-13 plasmid confers both carbenicillin and tetracycline resistance. Accuracy of spiking was initially determined by spot plating serial dilutions of the mixed culture at 1:10^5^ and 1:10^6^ ratios on carbenicillin or tetracycline containing plates, through which we were able to confirm that the anti-IL-13 antibody was represented in the pool at the anticipated frequency (Fig. 2A). For accurate quantification of the sorting experiments that followed, sort inputs and outputs were plated and colonies were counted to calculate the ratio of anti-IL-13 to anti-VEGF.1 in each sample. Starting with a 1:10^6^ ratio of anti-IL-13:anti-VEGF.1, we performed two rounds of BAD by sorting for the anti-IL-13-positive cells and achieved a 235,000-fold enrichment (Fig. 2B). To ensure we could enrich a separate antigen/antibody pair, we performed the converse experiment and saw a 19,500-fold enrichment of the binding anti-VEGF.1 antibody after two rounds of FACS (Fig. 2B). This enrichment corresponds to a large VEGF-positive shift in the population after the second round of FACS (Fig. 2C). These experiments confirm that binding clones can be rapidly and highly enriched by our BAD system.

**Figure 3:**
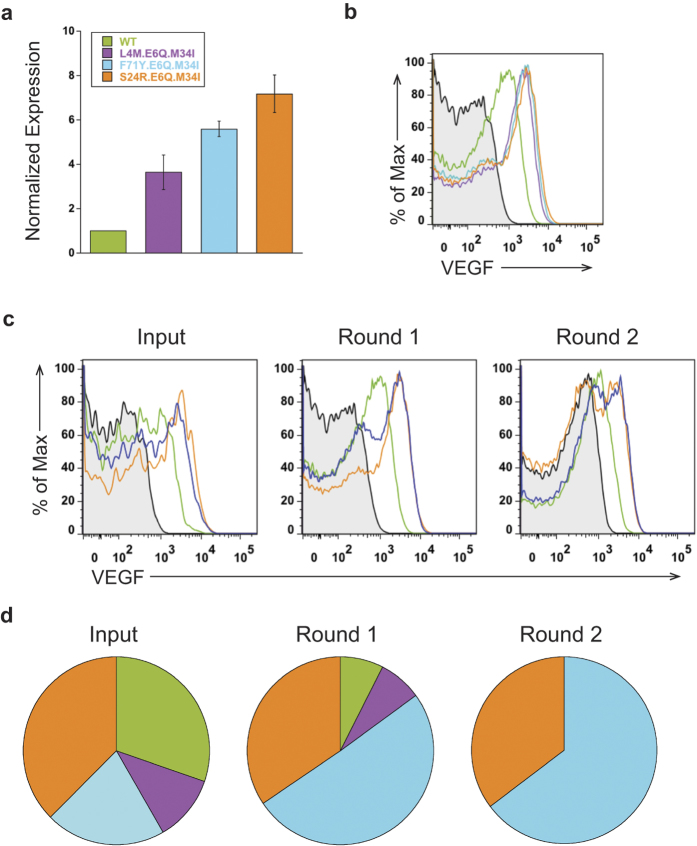
BAD resolves small differences in functional expression of anti-VEGF.2 antibody and three variants. (Color in panels **A–D**: anti-VEGF.2 WT (green), L4M.E6Q.M34I (purple), F71Y.E6Q.M34I (cyan), and S24R.E6Q.M34I (orange)). (**A**) Western blot expression levels of anti-VEGF.2 variants. Non-reduced soluble samples were detected with an anti-human Fc antibody and quantified using a LICOR Odyssey instrument. The signal was normalized to the half-antibody band of anti-VEGF.2 WT and n = 3 experiments. All variants have expression levels better than WT. (**B**) Representative flow cytometric analysis of individual anti-VEGF.2 variants. Anti-VEGF.2 WT has a higher shift than unstained cells (black, shaded). All variants have better shifts than WT, and correlate to the expression rankings seen by western blot (panel **A**). (**C**) Enrichment of best expressing variants from a pool of anti-VEGF.2 WT and three variants by BAD. The FACS signal of the pool (blue) shifts more positive over two rounds by sorting for the top 1% VEGF-binding cells. The lowest expressing anti-VEGF.2 WT (green) and the best expressing variant S24R.E6Q.M34I (orange) were analyzed in each round and are shown for comparison. (**D**) Anti-VEGF.2 variant enrichment over two rounds of sorting. 96 clones were sequenced, and the highest expressing anti-VEGF.2 variants, F71Y.E6Q.M34I (cyan) and S24R.E6Q.M34I (orange), are enriched after two rounds, while the two lowest expression variants are depleted.

Next, we investigated if we could enrich for the best expression or folding variants of the same antibody. To test the resolution of our system, we used a half-antibody based on anti-VEGF.2 antibody along with three variants that have different expression levels. The variants include different combinations of light chain (L4M, S24R, F71Y) and heavy chain (E6Q, M34I) mutations. Non-reduced soluble samples were detected with an anti-human Fc antibody by western blot and expression levels were normalized to anti-VEGF.2 half-antibody wild-type (WT) (Fig. 3A). Anti-VEGF.2 WT has the lowest functional expression, while L4M.E6Q.M34I, F71Y.E6Q.M34I, and S24R.E6Q.M34I have 3.7-, 5.6- and 7.2-fold increases in expression levels, respectively. To ensure that expression and FACS shift were in agreement, we used BAD to determine the individual FACS shifts for anti-VEGF.2 WT and variants stained with fluorescently labeled VEGF (Fig. 3B). The FACS mean fluorescence intensities ranked in the same order as the expression levels, with L4M.E6Q.M34I, F71Y.E6Q.M34I, and S24R.E6Q.M34I having 1.25 +/− 0.2, 1.37 +/− 0.2, and 1.4 +/− 0.3 fold-increases compared to WT anti-VEGF.2. Thus, these antibodies were good candidates for testing if BAD can select for the best expressing antibodies.

We performed a BAD screen combining anti-VEGF.2 WT with the three variants. Two rounds of FACS were performed and the top 1% of VEGF-binding clones was sorted in each round. The FACS shift of the four combined anti-VEGF antibodies was monitored over two rounds of sorting (Fig. 3C). We observe a bimodal distribution of cells in some of the samples, which we attribute to partial cell permeabilization and background autofluorescence of *E. coli*. The VEGF binding signal of the pooled variants (blue) overlays after two rounds of sorting with the best expression variant, S24R.E6Q.M34I (orange), suggesting that the better expressing antibodies are being enriched. The Input sequencing shows that all four anti-VEGF antibodies are present at the start of the sort, while anti-VEGF.2 WT and L4M.E6Q.M34I are partially depleted after Round 1 (Fig. 3D). After Round 2, anti-VEGF.2 WT and L4M.E6Q.M34I are not detected, and only F71Y.E6Q.M34I and S24R.E6Q.M34I are enriched. This data demonstrates that despite small expression differences BAD can successfully enrich for the best expressing antibodies.

In an effort to increase the functional expression of an antibody, we utilized the natural framework diversity that occurs during somatic hypermutation. We examined the Kabat database^23^ of naturally occurring antibodies and identified amino acid changes from germline in the framework regions of kappa 4 light chain and VH2 heavy chain subtypes, the same as our anti-IL-13 antibody (Supplemental Fig. 1). We reasoned that framework residues were less likely to affect affinity than the residues in the complementarity determining regions. Additionally, we focused on buried but not surface exposed residues based on a homology model, further hypothesizing that these could contribute to increased antibody folding and stability.

**Figure 4:**
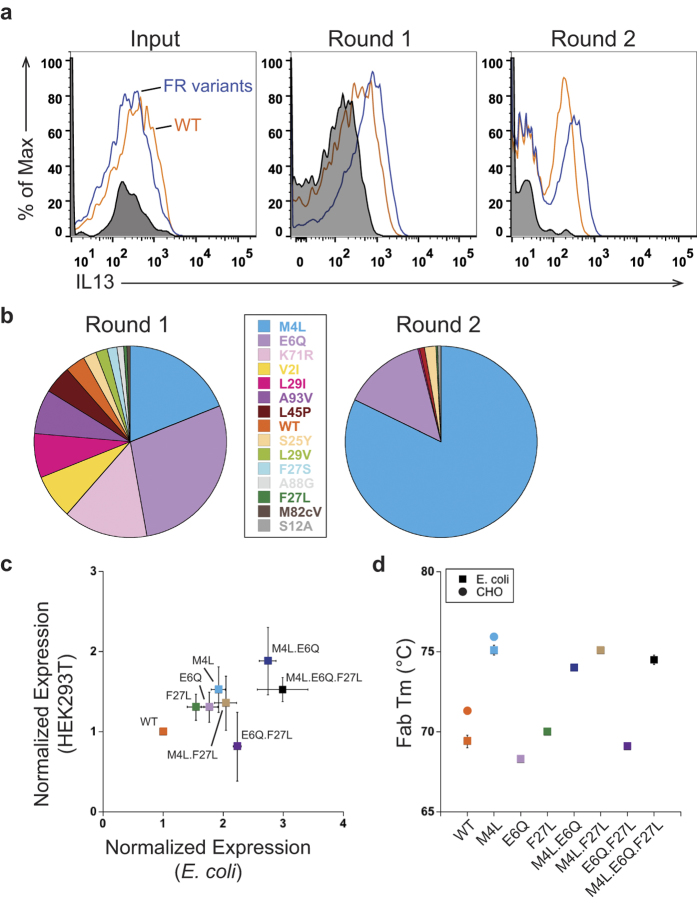
BAD identifies anti-IL-13 framework variants that increase functional expression from a library of natural diversity variants. (**A**) Round enrichment of anti-IL-13 framework variants. Overlaid FACS dot plots show that anti-IL-13 framework (FR) variants (blue) shift more positive over each round compared with anti-IL-13 wild-type (WT) (orange) and unstained cells (black, shaded). (**B**) Anti-IL-13 library round enrichment. Data displayed from Round 1 and Round 2 are from sequencing 186 and 226 clones, respectively. M4L and E6Q are the most enriched variants after two rounds of BAD. (**C**) Correlation of the expression of anti-IL-13 variants and their combinations in mammalian (HEK293T) and *E. coli* cells. Improvement in expression is seen in both host systems. For *E. coli* expression, non-reduced soluble half-antibody samples were detected with an anti-human Fc antibody and quantified using a LICOR Odyssey instrument. For HEK293T expression, the Protein A purified IgG yields were quantified. The expressions were normalized to anti-IL-13 WT and n = 3 experiments. (**D**) Thermostability of anti-IL-13 variants and their combinations measured by differential scanning fluorimetry (DSF). All variants containing M4L show a 5 °C increase in thermostability independent of the host expression system. To measure thermostability, Fabs and IgG were purified from *E. coli* and CHO cells, respectively. We report the values for the Fab transition.

We performed a BAD screen with 33 individual framework variants of the anti-IL-13 half-antibody, which included 5 light chain variants and 28 heavy chain variants, and the anti-IL-13 WT (Supplemental Fig. 2). Monitoring the FACS data over two rounds, the anti-IL-13 framework variants show an increase in binding to IL-13 antigen compared to anti-IL-13 WT (Fig. 4A). We sorted for the top 1% of IL-13 positive cells, and the sort outputs were sequenced. Anti-IL-13 WT is depleted by Round 2 (Fig. 4B), and the light chain M4L variant is largely enriched (82% of sequenced clones). In addition, the heavy chain variant E6Q (14% of sequenced clones) is also enriched, while the heavy chain variants S25Y, F27L, L29I and L45P and the light chain variant S12A are each observed in less than 2% of the sequenced clones.

To validate that the anti-IL-13 framework variants found after Round 2 increase functional expression, their expression levels in *E. coli* were compared using an anti-human Fc western blot. The light chain variant M4L shows the highest increase in expression, about 2-fold better than anti-IL-13 WT (Fig. 4C), correlating to the best enrichment by BAD. The heavy chain framework variants E6Q and F27L also have higher expression levels than anti-IL-13 WT (Fig. 4C), whereas the rest of the variants have similar expression to anti-IL-13 WT (data not shown). The western blot data correlates to the ranking seen with individual framework variants binding to IL-13 antigen, measured by FACS mean fluorescence intensity (data not shown). We used antigen concentrations well above the K_D_ in the FACS to minimize the contribution of affinity differences and ensure that expression levels were driving the identification of the anti-IL-13 framework variants. Binding kinetic data was collected and confirms that the anti-IL-13 framework variants M4L, E6Q, F27L and anti-IL-13 WT have comparable affinities (Table 1).

In previous reports mutations that increased antibody expression in *E. coli* did not translate to eukaryotic host systems^5^,^24^. We were interested to see if our screening strategy had identified variants that correlate to higher secretion levels in mammalian cells, allowing translatability of variant selection between expression hosts. Therefore, we examined the HEK 293T cell expression yields of the anti-IL-13 half-antibodies that provided an expression increase in *E. coli* (Fig. 4C). While the gains were slightly smaller in eukaryotic cells, we observed a linear correlation (R = 0.96, p = 0.04) in expression gains between the two hosts.

To investigate if the improvement in expression is correlated to an increase in conformational stability, we performed differential scanning fluorimetry (DSF). The melting temperatures of anti-IL-13 Fabs produced in *E. coli* with M4L, E6Q, or F27L mutations were compared to WT (Fig. 4D). The M4L variant increases thermal stability by about 5 °C (Tm of 75.1 ± 0.3 °C) compared to WT anti-IL-13 (Tm of 69.4 ± 0.4 °C). The E6Q and F27L mutations, however, do not yield a significant increase in thermodynamic stability. To verify that the increase in thermostability for the M4L variant is host independent, we produced anti-IL-13 as IgG in CHO cells. A similar 5 °C increase in thermostability was seen in the eukaryotic host.

We postulated that combining the anti-IL-13 variants identified by our BAD screen might be additive and further increase functional expression. Therefore, we examined expression levels in *E. coli* and mammalian cells for combinations of M4L, E6Q, and F27L (Fig. 4C). The double combination M4L.E6Q shows an additive increase in expression levels in both hosts. These two variants were the only variants largely enriched in our BAD screen, further supporting the selection capabilities of our technology. Additionally, only variant combinations containing the M4L mutation show a 5 °C increase in thermal stability (Fig. 4D). Thus, we identified a set of core framework variants that increase protein folding in multiple host systems and improve *in vitro* thermal stability of an anti-IL-13 antibody. While we improved the *in vitro* properties of the molecule, the clinical relevance of such variants has not been tested.

To further confirm that this strategy is generally applicable, we extended our studies to an antibody from a different germline, the anti-VEGF.3 antibody. Anti-VEGF.3 has kappa 1 light chain and VH3 heavy chain subtypes. Similar to our approach with anti-IL-13, naturally occurring antibodies with these distinct subtypes were collated from the Kabat database, and framework positions with variability from somatic hypermuation were identified (Supplemental Fig. 3). Positions known to be important for VEGF binding^25^ and those that are solvent exposed were excluded in the analysis to lower the impact on affinity or potential immunogenicity, respectively.

**Figure 5:**
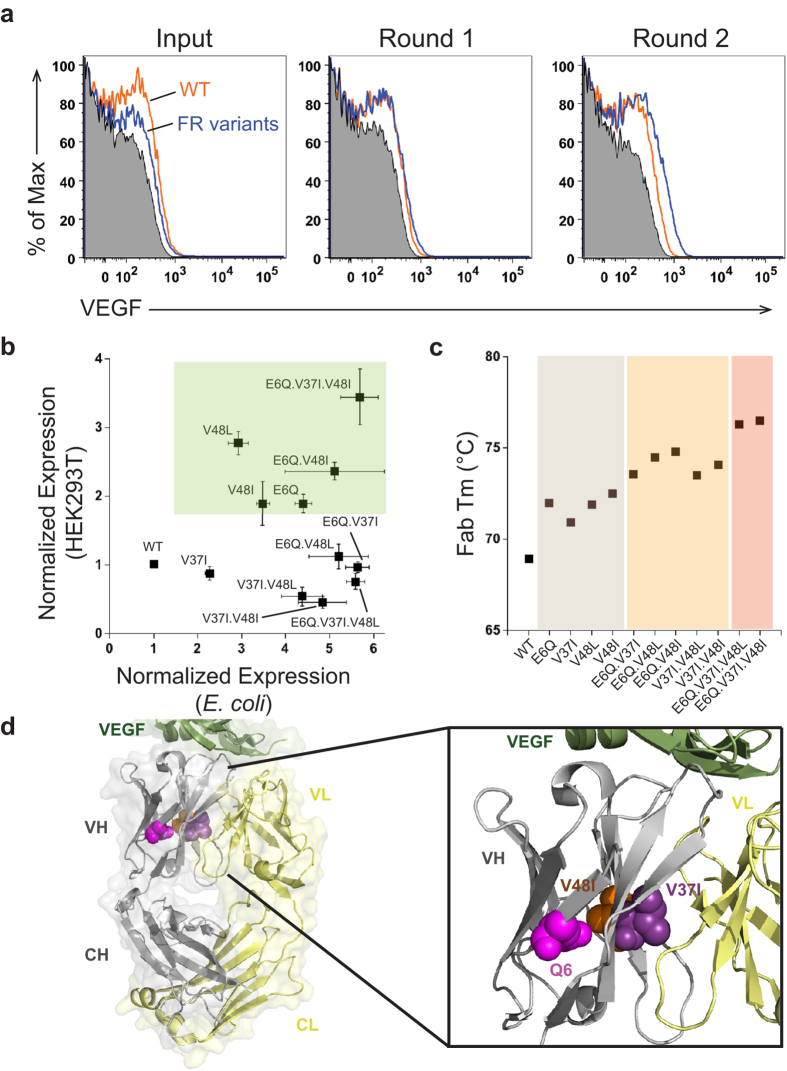
Anti-VEGF.3 framework variants with increased expression and thermostability are selected by BAD. (**A**) Anti-VEGF framework library enrichment is seen after each round of FACS. Flow cytometric analysis shows after Round 1 that anti-VEGF framework (FR) library variants (blue) shift better than anti-VEGF.3 wild-type (WT) (orange) or unstained cells (black, shaded), and the shift is further pronounced by Round 2. (**B**) A subset of the anti-VEGF framework variants and their combinations that improved expression in *E. coli* also show an increase in mammalian (HEK293T) expression (green panel). Expression was normalized to anti-VEGF WT and was determined by an anti-Fc western blot of half-antibody (*E. coli*) or Protein A purified IgG (HEK 293T), and n = 3 experiments. (**C**) Anti-VEGF.3 framework variants increase thermostability and their combinations have an additive effect. Differential scanning fluorimetry shows that the single framework variants E6Q, V37I, V48L or V48I (grey panel) increase thermostability. The double variants (orange panel) further increase thermostability, and the triple variants (salmon panel) have the best additive increases. Fab transition values of the IgG are reported with n = 3 experiments. (**D**) The identified framework residues which increase expression and thermostability are shown on the x-ray crystal structure of anti-VEGF G6 in complex with VEGF (PDB: 2FJG). V37I (purple) and V48I (orange) mutations were modeled, while Q6 (magenta) was in the native antibody structure. The three residues are located within a plane in the core of the variable heavy (VH) chain (grey). E6 and V48I are located on separate strands in the beta barrel core of the VH domain, while V37I is in the buried interface between the variable heavy and variable light (VL) chain (yellow). VEGF antigen (green), constant heavy (CH), constant light (CL).

We performed a BAD screen with the identified anti-VEGF framework variants, combining 47 light chain variants, 36 heavy chain variants, and anti-VEGF.3 WT. We sorted the top 0.5% VEGF-positive cells for three rounds. Anti-VEGF.3 WT is depleted by Round 1 as judged by sequencing of 192 output clones (data not shown) and framework variants show an increase in binding to VEGF antigen compared to anti-VEGF.3 WT with progressing rounds (Fig. 5A).

After Round 3, clones were enriched (Fig. 5A) and we confirmed the variants selected in BAD by expression in *E. coli* and HEK 293 T cells. Anti-VEGF heavy chain variants E6Q, V48L, and V48I (85%, 7%, and 3% of sequenced clones, respectively), improved expression by approximately 3-4.5-fold in *E. coli* (Fig. 5B). We next investigated if the improved expression is correlated to increased stability by performing DSF. All high expressing variants showed improved thermostability by about 3 °C compared to anti-VEGF.3 WT (Fig. 5C). The side chains of E6 and V48 (Fig. 5D) are positioned in the core of the beta-barrel of the variable heavy chain VEGF-Fab G6^26^. Since the E6Q or V48L/I variants improve folding and provide superior stability, it suggests that the beta-sheet interface is slightly underpacked, and removal of the charge or a larger side chain improve the packing. The V37I (1% of Round 3 sequenced clones) heavy chain variant improved the expression to a lesser extent (2.3-fold in *E. coli*) and increased thermostability by 2 °C (Fig. 5C). In contrast to E6 and V48, V37 is found at the interface with the light chain (Fig. 5D), illustrating the importance of the inter-chain as well as intra-chain contacts for antibody stability and folding.

Next we combined the framework variants to study the potential of additive effects. All double variants had additive effects with improvements on functional expression of up to 5.6-fold in *E. coli* (Fig. 5B), while the combination of the three variants did not further improve the expression of the anti-VEGF.3 antibody. Importantly, similar affinities to anti-VEGF.3 WT were observed for all single, double and triple variants (Table 2). To our surprise, additive effects were observed with all variant combinations on protein stability, and the wild-type Fab is successively further stabilized up to 7.6 °C by combining variants at all three positions (Fig. 5C). This impressive increase in thermal stability highlights how BAD technology can be used during therapeutic antibody development to identify variants with improved thermal stability.

When these variants and combinations were expressed in a mammalian system, several of the expression yields were consistent with the results observed in *E. coli* (Fig. 5B). All single variants, except V37I, improved the expression in HEK 293T cells by almost 2- to 3-fold. We saw an additional increase in expression by combining E6Q and V48I variants, and this was further improved by the V37I variant. The best expression was seen with this E6Q.V37I.V48I variant, which provided almost 4- or 6-fold increases in antibody yields in HEK 293T and *E. coli*, respectively.

In summary, this work demonstrates that the expression yields and conformational stability of an antibody can be significantly optimized by a few changes in the framework region without altering the antigen affinity. Even though these framework variants were initially identified in *E. coli* cells, expression increases translated to mammalian cells. This in turn supports the idea that the naturally occurring process of somatic hypermutation also enables B-cells to select for antibodies with improved stability and higher expression yields independent of antigen binding.

## Discussion

In this work, we establish a new library design based on the natural diversity found in the antibody framework region. Using this library in a newly developed live bacterial display system that does not require reformatting between selection rounds, we identify variant combinations that improve full-length antibody expression and stability in both *E. coli* and mammalian expression systems. This was enabled by the tight correlation between expression and thermostability in *E. coli*.

Previously, antibody expression and thermostability have been improved through the use of saturation mutagenesis^5^, rational mutagenesis^27^, error-prone PCR^28^, exchanging residues between framework subtypes^24^, or grafting the antibody binding residues onto a highly stable framework^29^. Contrary to these studies, we limited the number of framework mutations and focused on the natural diversity within a given subgroup, a novel approach for engineering a therapeutic antibody to reduce the immunogenicity risk, and maintain antigen-binding affinity. Furthermore, by restricting the residue changes to single, non-solvent exposed positions we designed a small, effective library that increased antibody yields and thermostability. This work further supports a recent study suggesting that framework mutations are introduced by somatic hypermutation to offset instability caused by affinity increasing mutations in the complementarity determining regions^30^. In addition, our work suggests that the majority of possible changes in the framework residues introduced during somatic hypermutation may have negative or neutral impact on antibody stability and expression, as only 5-10% of the single variants show improvements. However, combining these rare mutations can show additive effects to greatly improve expression and thermostability.

Most therapeutic proteins are produced in mammalian cells^31^, however there is a growing interest in using *E. coli* as an expression host for antibody or antibody-like proteins of therapeutic interest^21^,^32^,^33^,^34^. Therefore, it was desirable to establish a screening tool that can improve expression across host systems. In previous studies, variants that improved expression in *E. coli* did not improve expression in mammalian and yeast systems^5^,^24^. Our BAD system successfully identified framework variants that improve expression in both *E. coli* and mammalian systems. One reason for our success could be that we screen variants in *E. coli* using a full-length antibody format instead of using a Fab or scFv^5^,^24^. Additionally, our success could be based on our library design, which mimics the natural diversity introduced by somatic hypermutation rather than introducing non-natural changes. Previous work has shown that nature has already preselected these positions and changes to improve expression^30^.

More thermodynamically stable proteins often have higher yields when expressed in the periplasm of *E. coli*^5^,^35^ or in *Saccharomyces cerevisiae*^36^,^37^, and they also increase the long-term stability of proteins of therapeutic interest^38^,^39^. Thus, high thermostability is commonly a desired characteristic for a protein therapeutic. Most studies have focused on improving stability in proteins other than antibodies^36^,^37^, yet there are a few instances where antibodies were used. For example, the stability of an IgG1 Fc region was improved using yeast display combined with heat incubation. While similar gains in thermostability were observed compared to our method, the viability of the cells was sacrificed requiring PCR reformatting between selection rounds^28^. In addition, Fab and scFv stability were improved using *E. coli* and the gains translated from *E. coli* to yeast and mammalian expressed IgG^5^,^24^. In both approaches, multiple non-native changes were required to increase antibody stability. In our approach, a single variant from natural framework diversity can yield an equivalent or better increase in thermostability compared to previous methods.

During the discovery process, the framework region is often neglected due to the focus on residues important for antigen binding, thus leading to antibodies that may have suboptimal *in vitro* properties. Somatic mutations in hybridoma antibodies derived from wild-type or human transgenic animals are naturally selected by the B-cell for expression and antigen binding. Grafting CDRs into a germline or consensus framework, can leave behind the co-selected framework variants contributing to antibody stability and expression. Similarly during phage display or other *in vitro* selection methods, CDR-libraries may not be compatible with a standard framework, leading to antibody instability and aggregation^40^. Moreover, antibodies that display these poor *in vitro* characteristics can be lost during the selection process due to poor expression, decreasing the effective library size and excluding potential high-affinity binders. Here, we show that the use of framework libraries derived from naturally occurring somatic mutations can rescue antibody stability that is lost during the therapeutic discovery process. In addition to providing solutions to move antibodies into clinical development, it may be advantageous to include this framework diversity in the library design, allowing sampling of the full library diversity as well as selection of antibodies with improved *in vitro* properties.

Now that we have simplified the bacterial display process, it makes it an attractive tool for use in many applications. In the antibody community, it could be used with larger libraries for Fc-engineering, antibody discovery, or affinity maturation. The limitations of library size while screening with FACS could be overcome by using a pre-enriched phage library^41^. Beyond antibodies, our BAD system could be applicable as a general high throughput tool to study protein export, folding, or localization in the *E. coli* periplasm, as it offers a true readout in a cellular host environment. A major strength of our system is the non-tethered approach that now allows use of the native protein format, making fusion partners, reporter proteins, or membrane tethers unnecessary^42^,^43^. Furthermore, our live cell setting approach now enables the rapidly evolving field of genomic engineering to screen for desired host characteristics, something that was unattainable with previous display technologies.

## Materials and Methods

### Plasmid construction and expression

Antibodies were cloned by standard molecular biology techniques into single *E. coli* expression vectors with individual cistrons for light and heavy chain^1^,^44^ or separate mammalian expression vectors containing either light or heavy chain^45^,^46^. Expression of antibody genes in *E. coli* is controlled by the alkaline phosphatase promoter and targeted to the periplasm by the STII signal sequence. Expression of antibody genes in mammalian cells is controlled by the CMV promoter. Antibody expression was carried out using 50 mL *E. coli* cell cultures^21^ or 30 mL transient transfection cultures of CHO^47^ or HEK293T^48^ cells as previously described.

### Strain engineering

The ∆lpp deletion was transduced by P1-transduction^49^ into 62A7 (W3110 Δ*fhuA (ΔtonA) ptr3, lacIq, lacL8, ompTΔ(nmpc-fepE) ΔdegP ilvG*^*+*^) and 64B4 (W3110 Δ*fhuA (ΔtonA) ΔphoA ilvG2096 (ilvG*^*+*^*; Val*^*r*^*) ΔmanA Δprc spr43H1 ΔdegP lacI*^*q*^*ΔompT*)^50^ from JW1667 (Δ*(araD-araB)567 ΔlacZ4787*(::rrnB-3) *λ*^*−*^*Δlpp-752::kan rph-1 Δ(rhaD-rhaB)568 hsdR514*) (obtained from the Coli Genetic Stock Center, Yale)^51^. Strains were cured of kanamycin resistance by λ RED recombinase^52^ to obtain 66C4 and 65G4, respectively.

### Antibody purification

For purification of Fab protein from *E. coli*, protein was expressed as described earlier^20^ in strain 65G4. After growth of a 500 mL culture in completely radical alkaline phosphatase medium (CRAP)^1^,^53^ for 24 hours at 30 °C, EDTA pH 8.0 was added to 10 mM final concentration. Incubation continued for 1 hr at 30 °C at 200 rpm, before MgCl_2_ was added to 20 mM final concentration. Cell debris was removed by centrifugation (20 minutes, 4,500 rcf, 4 °C), 400 μg Desoxyribonuclease I (Sigma-Aldrich, USA) added before the supernatant was filtered through a GF/F filter (Whatman, England) and 0.2 μ PES filter (Thermo Fischer Scientific, USA). 1.5 mL Protein G slurry (GE Healthcare, USA) was added to 50 mL lysate and incubated overnight at room temperature. Unbound proteins were removed by washing with PBS, Fab protein was eluted with 4 ml 50 mM phosphoric acid pH 3, neutralized with 20 x PBS, and concentrated with an Amicon Ultra-4 concentrator (10,000 MW cutoff) (Merck Millipore, Ireland).

Human IgG1 was purified from mammalian culture supernatants by MabSelectSure (GE Healthcare, USA) according to the manufacturers protocol. For human Fab purification from mammalian culture supernantants, a FLAG tag was added to the C-terminus of the heavy chain and the Fab was purified with anti-FLAG resin according to the manufacturers protocol (Sigma-Aldrich, USA). Protein yields were quantified by determining the absorbance at 280nm and using the protein specific extinction coefficient.

### Antigens

Expression and purification of human VEGF_8-109_ was described previously^54^. Unizyme tagged human IL-13_33-146_ was expressed in *E. coli*, purified by Ni-NTA chromatography (Qiagen, Germany) under denaturing conditions and refolded. Proteins were fluorescently labeled with Alexa Fluor 488 or Alexa Fluor 647 succinimidyl ester (Invitrogen, USA) according to the manufacturers protocol. DyLight^649^ labeled goat anti-human IgG Fc F(ab’)_2_-fragment was obtained from Jackson Immuno Research, USA.

### Bacterial Antibody Display and FACS

For bacterial display, antibodies were expressed in a 66C4 cured of kanamycin resistance. For spiking experiments or experiments with individual framework variants, cells were individually transformed with the respective plasmids, grown as overnight cultures at 30 °C, combined in the ratios described or equally by volume and used at a 1:100 dilution to inoculate 50 mL CRAP cultures. After 24 hours at 30 °C, 1 OD aliquots were harvested and pelleted by centrifugation (4 minutes, 6500 rcf). Cells were resuspended in 100 μL PBS with 2% BSA and 5mM EDTA and incubated at 4 °C for 30 minutes. After initial incubation, SYTO 41 or 9 nucleic acid stain (Molecular Probes, USA) was added to a final dilution of 1:100, and Alexa^488^ or Alexa^647^ labeled antigen added to a final concentration of 1–2 μM. Incubation was continued at 4 °C in darkness for 1 hour, at which time MgCl_2_ was added to 10-20 mM final concentration. Unbound proteins were removed by washing 3 times with 1 mL volumes of PBS + 20 mM MgCl_2_. Stained cells were resuspended in SOC medium (New England Biolabs, USA) to a final concentration of 1 × 10^7^ cells/ml for analysis and 1 × 10^6^ cells/ml for sorting using a Becton Dickenson FACS AriaII Flow Cytometer. The data was processed using FlowJo and an automated scaling mode was used that normalizes the number of cells. The FACS gating strategy included cells that were SYTO dye-positive. Doublet discrimination gates were used to remove doublets and finally antigen-positive cells were sorted. Cells were sorted into SOC media, recovered at 30 °C overnight, and used to reinoculate expression cultures or frozen as glycerol stocks.

The cell populations that are shown in the histograms are the total number of cells that stain positive for DNA dye, which include antigen-positive and antigen-negative cells. The signal from the large number of antigen-negative cells goes to 100% in the histograms, however it is difficult to see because many of them are close to the y-axis.

### Microscopy

Expression and staining of cells for fluorescent microscopy was performed identically to the bacterial display protocol described above. Following the PBS with 20 mM MgCl_2_ wash steps, cells were resuspended in 100 μL PBS with 20 mM MgCl_2_ and mixed with 1% gelatin at 37 °C for slide preparation. Fluorescent microscopy was performed using a Zeiss Axio Imager A1.

### Immunoblots

Immunoblotting was performed as described previously^20^, except antibodies were expressed in 62A7.

### Protein stability measurements by differential scanning fluorimetry

Protein stability was determined in a Biorad CFX96 Real-Time System (Biorad, USA) with a final dilution of 1:500 of the Sypro Orange dye stock (Molecular Probes, USA). Fluorescence of a 25 μL sample (0.1 mg/ml) in PBS was recorded from 20–100 °C (0.2 °C increments, 10 seconds hold per step). The assignment of the Fab melt was done as published previously^55^, the C_H_2 and C_H_3 domains had consistent transitions across the variants.

### Periplasmic Extract

To generate periplasmic extract for Biacore analysis, 40 mL of *E. coli* expression broth, from the bacterial antibody display strain, was pelleted by centrifugation at 5,000 rpm for 5 minutes. Pellets were resuspended in 1.5 mL of cold 50 mM Tris pH 8.0, 1 mM EDTA and 500 mM sucrose and incubated on ice for 30 min with shaking. After centrifugation of the sample at 9000 rpm for 10 min, the supernatant was collected as the periplasmic extract.

### Biacore

The binding kinetics of the anti-IL-13 and anti-VEGF antibodies was measured using surface plasmon resonance on a Biacore 3000 or T200 instrument (GE Healthcare), respectively. All kinetics experiments were performed at a flow rate of 30 μL/min, at 25 °C, and with a running buffer of 10 mM HEPES, pH 7.4, 150 mM NaCl, and 0.005% Tween 20. For anti-IL-13, Fab Binder from the Human Fab Capture Kit (GE Healthcare) was immobilized on a CM5 sensor chip via amine-based coupling to capture anti-IL-13 half antibodies from *E. coli* periplasmic extracts. Human IL-13 binding to the antibody was measured using a two-fold concentration series of cytokine with a range of 1.56 to 50 nM. Sensorgrams for binding of IL-13 were recorded using an injection time of 120 seconds followed by 1000 seconds of dissociation time and regeneration of the surface between cycles with glycine pH 2.1. For anti-VEGF, human VEGF was immobilized on a CM5 sensor chip via amine-based coupling. A three-fold concentration series of anti-VEGF Fabs ranging from 6.17 to 500 nM was used to analyze binding to VEGF. Single cycle kinetics sensorgrams were recorded using an injection time of 120 seconds followed by 300 seconds of dissociation time and regeneration of the surface between cycles with 20 mM HCl. All sensorgrams observed for antigen binding to antibodies were analyzed using a 1:1 Langmuir binding model to calculate the kinetics and binding constants.

## Additional Information

**How to cite this article**: Lombana, T. N. *et al.* Optimizing antibody expression by using the naturally occurring framework diversity in a live bacterial antibody display system. *Sci. Rep.* **5**, 17488; doi: 10.1038/srep17488 (2015).

## Supplementary Material

Supplementary Information

## Figures and Tables

**Table 1:** Antigen affinity of anti-IL-13 half-antibody framework variants.

	k_a_ (1/Ms, E + 5)	k_d_ (1/s, E-5)	K_D_ (nM)
WT	13.0 ± 0.700	5.09 ± 1.40	0.040 ± 0.013
M4L	11.1 ± 0.603	3.16 ± 1.12	0.029 ± 0.010
E6Q	11.3 ± 1.01	4.26 ± 0.445	0.038 ± 0.007
F27L	13.0 ± 0.954	4.43 ± 0.362	0.034 ± 0.003

All experiments were performed 3 times.

**Table 2:** Antigen affinity of anti-VEGF.3 Fab framework variants.

	k_a_ (1/Ms, E + 5)	k_d_ (1/s, E-5)	K_D_ (nM)
WT	0.313 ± 0.016	47.1 ± 1.94	15.1 ± 1.17
E6Q	0.448 ± 0.011	59.2 ± 3.71	13.2 ± 1.05
V37I	0.330 ± 0.014	50.8 ± 3.75	15.4 ± 1.79
V48L	0.346 ± 0.024	47.1 ± 3.26	13.6 ± 1.21
V48I	0.319 ± 0.011	47.1 ± 2.61	14.8 ± 1.25
E6Q.V37I	0.428 ± 0.008	57.7 ± 2.75	13.5 ± 9.17
E6Q.V48L	0.457 ± 0.013	50.6 ± 2.66	11.1 ± 9.07
E6Q.V48I	0.421 ± 0.013	56.6 ± 2.60	13.5 ± 1.06
V37I.V48L	0.352 ± 0.015	45.0 ± 2.82	12.8 ± 1.37
V37I.V48I	0.335 ± 0.014	50.5 ± 3.32	15.1 ± 1.61
E6Q.V37I.V48L	0.471 ± 0.079	53.0 ± 2.75	11.5 ± 2.21
E6Q.V37I.V48I	0.414 ± 0.015	58.4 ± 3.42	14.2 ± 1.36

All experiments were performed 3 times.

## References

[b1] Simmons L. C. *et al.* Expression of full-length immunoglobulins in Escherichia coli: rapid and efficient production of aglycosylated antibodies. J Immunol Methods 263, 133–147 (2002). 12009210 10.1016/s0022-1759(02)00036-4

[b2] Chen C. *et al.* High-level accumulation of a recombinant antibody fragment in the periplasm of Escherichia coli requires a triple-mutant (degP prc spr) host strain. Biotechnol Bioeng 85, 463–474 (2004). 14760686 10.1002/bit.20014

[b3] Makino T., Skretas G., Kang T. H. & Georgiou G. Comprehensive engineering of Escherichia coli for enhanced expression of IgG antibodies. Metab. Eng. 13, 241–251 (2011). 21130896 10.1016/j.ymben.2010.11.002 PMC3057344

[b4] Bothmann H. & Plückthun A. The periplasmic Escherichia coli peptidylprolyl cis,trans-isomerase FkpA. I. Increased functional expression of antibody fragments with and without cis-prolines. J Biol Chem 275, 17100–17105 (2000). 10748200 10.1074/jbc.M910233199

[b5] Demarest S. J. *et al.* Engineering stability into Escherichia coli secreted Fabs leads to increased functional expression. Protein Eng Des Sel 19, 325–336 (2006). 16672248 10.1093/protein/gzl016

[b6] Zhai W. *et al.* Synthetic Antibodies Designed on Natural Sequence Landscapes. J Mol Biol 412, 55–71 (2011). 21787786 10.1016/j.jmb.2011.07.018

[b7] Boder E. T. & Wittrup K. D. Yeast surface display for screening combinatorial polypeptide libraries. Nat Biotechnol 15, 553–557 (1997). 9181578 10.1038/nbt0697-553

[b8] Feldhaus M. J. *et al.* Flow-cytometric isolation of human antibodies from a nonimmune Saccharomyces cerevisiae surface display library. Nat Biotechnol 21, 163–170 (2003). 12536217 10.1038/nbt785

[b9] Zhou C., Jacobsen F. W., Cai L., Chen Q. & Shen W. D. Development of a novel mammalian cell surface antibody display platform. MAbs 2, 508–518 (2010). 20716968 10.4161/mabs.2.5.12970 PMC2958572

[b10] Löfblom J. Bacterial display in combinatorial protein engineering. Biotechnol J 10.1002/biot.201100129 (2011). 21786423

[b11] Daugherty P. S., Olsen M. J., Iverson B. L. & Georgiou G. Development of an optimized expression system for the screening of antibody libraries displayed on the Escherichia coli surface. Protein Eng. 12, 613–621 (1999). 10436088 10.1093/protein/12.7.613

[b12] Daugherty P. S. Protein engineering with bacterial display. Curr. Opin. Struct. Biol. 17, 474–480 (2007). 17728126 10.1016/j.sbi.2007.07.004

[b13] Rockberg J., Löfblom J., Hjelm B., Uhlén M. & Ståhl S. Epitope mapping of antibodies using bacterial surface display. Nat. Methods 5, 1039–1045 (2008). 19029907 10.1038/nmeth.1272

[b14] Chen G. *et al.* Isolation of high-affinity ligand-binding proteins by periplasmic expression with cytometric screening (PECS). Nat Biotechnol 19, 537–542 (2001). 11385457 10.1038/89281

[b15] Dodevski I. & Plückthun A. Evolution of Three Human GPCRs for Higher Expression and Stability. J Mol Biol 408, 599–615 (2011). 21376730 10.1016/j.jmb.2011.02.051

[b16] Mazor Y., Van Blarcom T., Mabry R., Iverson B. L. & Georgiou G. Isolation of engineered, full-length antibodies from libraries expressed in Escherichia coli. Nat Biotechnol 25, 563–565 (2007). 17435747 10.1038/nbt1296

[b17] Leive L., Shovlin V. K. & Mergenhagen S. E. Physical, chemical, and immunological properties of lipopolysaccharide released from Escherichia coli by ethylenediaminetetraacetate. J Biol Chem 243, 6384–6391 (1968). 4973230

[b18] Hancock R. E. W. Alterations in Outer Membrane Permeability. Annu Rev Microbiol 38, 237–264 (1984). 6093683 10.1146/annurev.mi.38.100184.001321

[b19] Kanamori T. *et al.* Expression and excretion of human pancreatic secretory trypsin inhibitor in lipoprotein-deletion mutant of Escherichia coli. Gene 66, 295–300 (1988). 3049250 10.1016/0378-1119(88)90365-4

[b20] Spiess C. *et al.* Bispecific antibodies with natural architecture produced by co-culture of bacteria expressing two distinct half-antibodies. Nat Biotechnol 31, 753–758 (2013). 23831709 10.1038/nbt.2621

[b21] Spiess C. *et al.* Development of a Human IgG4 Bispecific Antibody for Dual Targeting of Interleukin-4 (IL-4) and Interleukin-13 (IL-13) Cytokines. Journal of Biological Chemistry 288, 26583–26593 (2013). 23880771 10.1074/jbc.M113.480483 PMC3772205

[b22] Junttila T. T. *et al.* Antitumor Efficacy of a Bispecific Antibody That Targets HER2 and Activates T Cells. Cancer Res. 74, 5561–5571 (2014). 10.1158/0008-5472.CAN-13-3622-T 25228655

[b23] Kabat E. A., Wu T. T., Perry H., Gottesman K. & Foeller C. Sequences of proteins of immunological interest. NIH Publication (1991).

[b24] Schaefer J. V. & Plückthun A. Transfer of engineered biophysical properties between different antibody formats and expression systems. Protein Engineering Design and Selection 25, 485–506 (2012). 10.1093/protein/gzs039 22763265

[b25] Presta L. G. *et al.* Humanization of an anti-vascular endothelial growth factor monoclonal antibody for the therapy of solid tumors and other disorders. Cancer Res. 57, 4593–4599 (1997). 9377574

[b26] Fuh G. Structure-Function Studies of Two Synthetic Anti-vascular Endothelial Growth Factor Fabs and Comparison with the AvastinTM Fab. Journal of Biological Chemistry 281, 6625–6631 (2006). 16373345 10.1074/jbc.M507783200

[b27] Wall J. G. & Plückthun A. The hierarchy of mutations influencing the folding of antibody domains in Escherichia coli. Protein Eng. 12, 605–611 (1999). 10436087 10.1093/protein/12.7.605

[b28] Traxlmayr M. W. *et al.* Directed evolution of stabilized IgG1-Fc scaffolds by application of strong heat shock to libraries displayed on yeast. Biochim. Biophys. Acta 1824, 542–549 (2012). 22285845 10.1016/j.bbapap.2012.01.006 PMC3787792

[b29] Willuda J. *et al.* High thermal stability is essential for tumor targeting of antibody fragments: engineering of a humanized anti-epithelial glycoprotein-2 (epithelial cell adhesion molecule) single-chain Fv fragment. Cancer Res. 59, 5758–5767 (1999). 10582696

[b30] Wang F. *et al.* Somatic hypermutation maintains antibody thermodynamic stability during affinity maturation. Proc Natl Acad Sci USA 110, 4261–4266 (2013). 23440204 10.1073/pnas.1301810110 PMC3600448

[b31] Zhang R. & Shen W. In (ed. Chames P.) 907, 341–358 (Humana Press, 2012). 10.1007/978-1-61779-974-7_20 22907362

[b32] Merchant M. *et al.* Monovalent antibody design and mechanism of action of onartuzumab, a MET antagonist with anti-tumor activity as a therapeutic agent. Proc. Natl. Acad. Sci. USA 110, E2987–E2996 (2013). 23882082 10.1073/pnas.1302725110 PMC3740879

[b33] Chen Y. *et al.* Selection and analysis of an optimized anti-VEGF antibody: crystal structure of an affinity-matured fab in complex with antigen. J Mol Biol 293, 865–881 (1999). 10543973 10.1006/jmbi.1999.3192

[b34] Stumpp M. T. & Amstutz P. DARPins: a true alternative to antibodies. Curr Opin Drug Discov Devel 10, 153–159 (2007). 17436550

[b35] Kwon W. S., Da Silva N. A. & Kellis J. T. Relationship between thermal stability, degradation rate and expression yield of barnase variants in the periplasm of Escherichia coli. Protein Eng. 9, 1197–1202 (1996). 9010933 10.1093/protein/9.12.1197

[b36] Traxlmayr M. W. & Obinger C. Directed evolution of proteins for increased stability and expression using yeast display. Arch Biochem Biophys 526, 174–180 (2012). 22575387 10.1016/j.abb.2012.04.022

[b37] Angelini A. *et al.* Protein Engineering and Selection Using Yeast Surface Display. Methods Mol Biol 1319, 3–36 (2015). 26060067 10.1007/978-1-4939-2748-7_1

[b38] Chaudhuri R., Cheng Y., Middaugh C. R. & Volkin D. B. High-throughput biophysical analysis of protein therapeutics to examine interrelationships between aggregate formation and conformational stability. AAPS J 16, 48–64 (2014). 24174400 10.1208/s12248-013-9539-6 PMC3889527

[b39] Asial I. *et al.* Engineering protein thermostability using a generic activity-independent biophysical screen inside the cell. Nature Communications 4, 2901 (2013). 10.1038/ncomms3901 24352381

[b40] Bradbury A. R. M., Sidhu S., Dübel S. & McCafferty J. Beyond natural antibodies: the power of *in vitro* display technologies. Nat Biotechnol 29, 245–254 (2011). 21390033 10.1038/nbt.1791 PMC3057417

[b41] Ferrara F. *et al.* Using Phage and Yeast Display to Select Hundreds of Monoclonal Antibodies: Application to Antigen 85, a Tuberculosis Biomarker. PLoS ONE 7, e49535 (2012). 23166701 10.1371/journal.pone.0049535 PMC3498134

[b42] Quan S. *et al.* Genetic selection designed to stabilize proteins uncovers a chaperone called Spy. Nature structural & molecular biology 18, 262–269 (2011). 10.1038/nsmb.2016 PMC3079333 21317898

[b43] Dinh T. & Bernhardt T. G. Using superfolder green fluorescent protein for periplasmic protein localization studies. J. Bacteriol. 193, 4984–4987 (2011). 21764912 10.1128/JB.00315-11 PMC3165667

[b44] Carter P., Kelley R. F., Rodrigues M. L. & Snedecor B. High level Escherichia coli expression and production of a bivalent humanized antibody fragment. Nature 10.1038/nbt0292-163 (1992). 1368228

[b45] Eaton D. L. *et al.* Construction and characterization of an active factor VIII variant lacking the central one-third of the molecule. Biochemistry 25, 8343–8347 (1986). 3030393 10.1021/bi00374a001

[b46] Polson A. G. *et al.* Antibody-drug conjugates targeted to CD79 for the treatment of non-Hodgkin lymphoma. Blood 110, 616–623 (2007). 17374736 10.1182/blood-2007-01-066704

[b47] Wong A. W., Baginski T. K. & Reilly D. E. Enhancement of DNA uptake in FUT8‐deleted CHO cells for transient production of afucosylated antibodies. Biotechnol Bioeng 106, 751–763 (2010). 20564613 10.1002/bit.22749

[b48] Bos A. B. *et al.* Development of a semi-automated high throughput transient transfection system. Journal of Biotechnology 180, 10–16 (2014). 24704608 10.1016/j.jbiotec.2014.03.027

[b49] Sternberg N. & Hoess R. The molecular genetics of bacteriophage P1. Annu. Rev. Genet. 17, 123–154 (1983). 6364958 10.1146/annurev.ge.17.120183.001011

[b50] Veeravalli K., Laird M. W., Fedesco M., Zhang Y. & Yu X. C. Strain engineering to prevent norleucine incorporation during recombinant protein production in Escherichia coli. Biotechnol. Prog. 31, 204–211 (2015). 25315437 10.1002/btpr.1999

[b51] Baba T. *et al.* Construction of Escherichia coli K-12 in-frame, single-gene knockout mutants: the Keio collection. Mol. Syst. Biol. 2, 2006.0008 (2006). 10.1038/msb4100050 PMC1681482 16738554

[b52] Datsenko K. A. & Wanner B. L. One-step inactivation of chromosomal genes in Escherichia coli K-12 using PCR products. Proc Natl Acad Sci USA 97, 6640–6645 (2000). 10829079 10.1073/pnas.120163297 PMC18686

[b53] Reilly D. E. & Yansura D. G. Antibody Engineering. 331–344 10.1007/978-3-642-01147-4_26 (Springer: Berlin Heidelberg,, 2010).

[b54] Muller Y. A. *et al.* Vascular endothelial growth factor: crystal structure and functional mapping of the kinase domain receptor binding site. Proc Natl Acad Sci USA 94, 7192–7197 (1997). 9207067 10.1073/pnas.94.14.7192 PMC23789

[b55] Ionescu R. M., Vlasak J., Price C. & Kirchmeier M. Contribution of variable domains to the stability of humanized IgG1 monoclonal antibodies. J Pharm Sci 97, 1414–1426 (2008). 17721938 10.1002/jps.21104

