# Supplementary material for: Optimizing antibody expression by using the naturally occurring framework diversity in a live bacterial antibody display system
Source: Sci Rep. 2015 Dec 3;5:17488. doi: 10.1038/srep17488 (PMC4668361; doi:10.1038/srep17488)
Supplement: Supplementary Information [file srep17488-s1.pdf]

Optimizing antibody expression by using the naturally occurring framework diversity in a live bacterial antibody display system

T. Noelle Lombana[1,2], Michael Dillon[1,2], Jack Bevers III[1], Christoph Spiess[1, 3]

Department of Antibody Engineering[1], Genentech Research and Early Development, 1 DNA Way, South San Francisco, CA, 94080, USA.

[2] These authors contributed equally to this work.

[3] Correspondence should be addressed to christsp@gene.com (phone +1 650 467 1851, fax +1 650 467 8318)

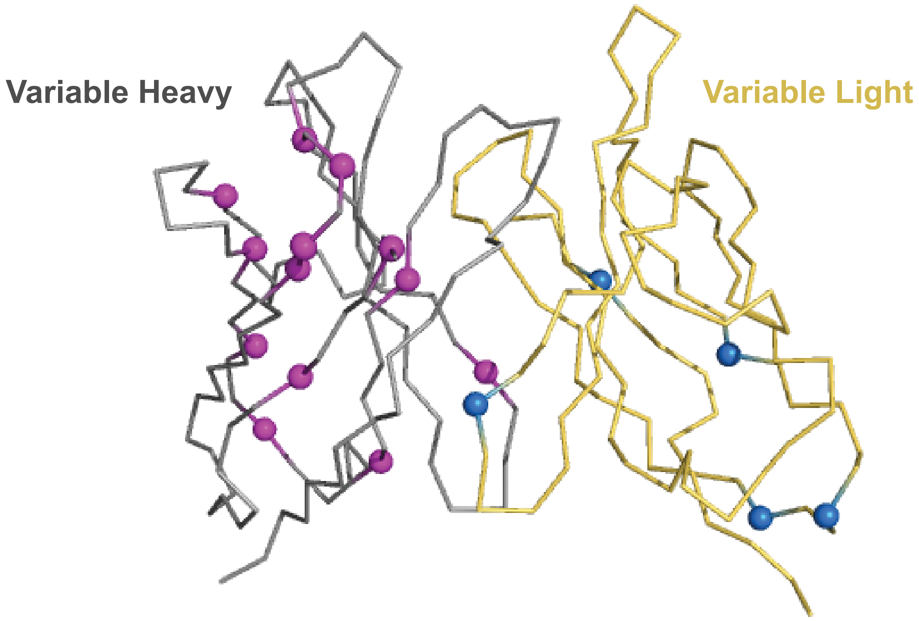

Heavy Chain Subgroup 2

Kabat #	Mutations
2	I, L
6	Q
24	F, L
25	T, Y
27	S, D, L, G
29	V, F, I
45	P
71	R, I, L, V
78	F
82	V, L
82c	V
88	G, P
93	T, N, V

Kappa Light Chain Subgroup 4

Kabat #	Mutations
4	L
12	A
15	P
21	V
43	A

Supplementary Figure 1. Ribbon diagram of the main chain antibody backbone based on the subgroup of the anti-IL-13 variable domains. Somatic hypermutation can introduce changes into both the framework regions and complementarity determining regions. Collating data from the Kabat database, we compiled all the amino acid changes that occur by somatic hypermutation within the framework of the Variable Kappa 4 (V_K4, yellow) and Heavy 2 (V_H2, grey) subtypes, then narrowed our chosen variants to the non-solvent exposed residues (spheres). We made single framework variants of anti-IL-13 half-antibody, 5 in light chain and 28 in heavy chain, and combined these to screen by BAD.

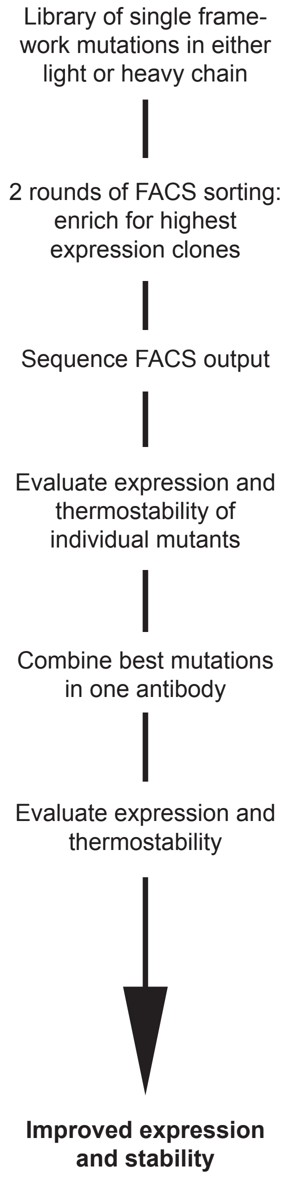

Supplementary Figure 2. Sequence of experiments identifying mutations in the antibody heavy and light chain that improve expression and stability. First, single framework mutations in either light or heavy chain were sorted by BAD for highest expression clones. After validating the improved expression by western blot, the mutations were combined to identify additive effects on expression and thermostability.

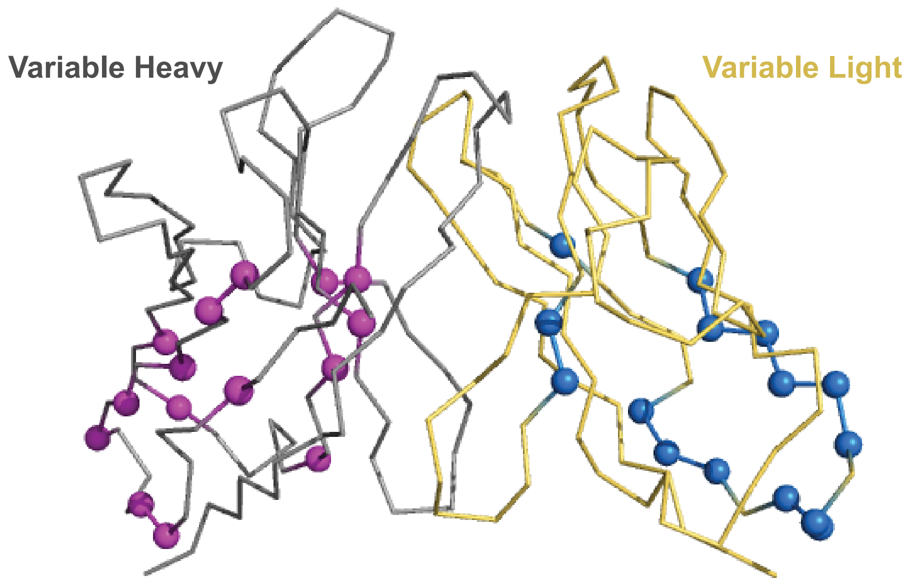

Heavy Chain Subgroup 3

Kabat #	Mutations
6	G, Q, D
11	V, F, S
12	I
16	R, T
18	M, G, R
20	V
23	T, V, E, S
24	S, T, V, G
37	L, I, F
48	L, I
82	L, I
82c	G, V
87	M, S
88	G
91	F
93	V, T

Kappa Light Chain Subgroup 1

Kabat #	Mutations	Kabat #	Mutations
4	L, V	58	I, A
9	T	62	V
10	T, F	64	D
11	V, Q, F	66	E, I, R
13	V, T	71	T, V
14	T, Y, V, F	73	F, V
15	T	75	V
17	G	78	V
18	S, T		
19	I		
20	A, I		
21	L, F, V		
22	I, S, A, R		
36	F		
37	D, E, H		

Supplementary Figure 3. Ribbon diagram of the main chain antibody backbone for the variable domains of the anti-VEGF G6 antibody [26] light chain (yellow) and heavy chain (grey). Similar to our approach with the anti-IL-13 antibody (see Supplementary Fig. 1), we scanned the Kabat database to identify residues introduced by somatic hypermutation that were in framework regions (FR) of Variable Kappa 1 and Heavy 3 (V_K1 and V_H3, respectively) subtypes. We chose to exclude the complementarity determining regions to maintain affinity while also selecting for buried residues (spheres) to reduce the risk of immunogenicity. With these criteria, we created single framework variants of anti-VEGF half-antibody, 47 in light chain and 36 in heavy chain, and combined these to screen by BAD.
